# Acute postoperative complications of hypospadias repair

**DOI:** 10.4103/0970-1591.40622

**Published:** 2008

**Authors:** Amilal Bhat, Arup Kumar Mandal

**Affiliations:** Department of Urology, S.P. Medical College, Bikaner, Rajasthan, India; 1 Postgraduate Institute of Medical Education and Research, Chandigarh, India

**Keywords:** Acute complications, hypospadias repair, management

## Abstract

**Purpose:**

Complications in hypospadias surgery are higher than other reconstructive procedures. The incidence of complications can be reduced if proper preventive measures are taken. The review aims to highlight incidences, causes, and preventive measures of acute complications of hypospadias repair.

**Materials and Methods:**

Literature reports were reviewed in Pubmed by giving the key word acute complications of hypospadias repair, wound infection, wound dehiscence, flap necrosis, edema, penile torsion, urethral fistula, bleeding and hematoma and urethral stents problems. Summaries of all articles were reviewed with full text of relevant article and results were analyzed.

**Results:**

Besides mentioning the complications of hypospadias repair in individual articles on the subject, we did not come across any separate article on this subject in the published English literature. Fistula is the commonest complication followed by edema and penile torsion.

**Conclusions:**

Most acute complications can be prevented with adherence to principles of plastic and microsurgery, meticulous preoperative planning, and judicious postoperative care. Deviation from these principles may lead to disaster and even failure of the repair. The aim in hypospadias surgery should be following these principles and bring down the complication rates < 5% in distal hypospadias and < 10% in proximal hypospadias.

Hypospadias is defined as a incomplete virilization of the genital tubercle leading to an ectopic opening of the urethra on the ventral aspect of the penis, any where from the glans to the perineum with or without ventral curvature and a ventral prepucial defect. Surgery is the only modality of the treatment. The aim of surgery is to achieve a straight penis, with the meatus at the tip, uninterrupted urinary flow, good cosmesis, and self-confidence of the child. Among the choice of procedures for distal hypospadias are plate preservation procedures like incised plate urethroplasty, glans approximation procedures, and MAGPAI whereas for proximal hypospadias is extended application of incised plate urethroplasty, various flap, and graft urethroplasties in one or two stages. Technically all flaps have a better blood supply than the grafts. The results are better with flap urethroplasties than grafts.[1] Rebirth of two-stage procedures with reduced complication rates has given a wider choice of procedures to the hypospadialogist for repair of proximal hypospadias. Complications after surgical procedures are not infrequent. Description of over 250 procedures for hypospadias repair suggests lack of uniformity of results of repair and high-complication rates. Acute complications occur within 7-10 days following surgery and require a proper assessment and decision making for management. Mismanagement can result in failure of the procedure. Complication rates are higher in onlay flap or inner prepucial tube as compared to plate preservation procedures like incised plate urethroplasty and in two-stage urethroplasty as compared to one stage. A proper preoperative assessment and planning is must for good results. A mistaken attempt to apply a minor hypospadias repair to a major deformity would lead to complications and failure. The purpose of this review was to evaluate causes, incidences, and preventive measures of the acute complications of hypospadias repair from the literature and from our own experience.

## MATERIALS AND METHODS

Literature reports were reviewed in Pubmed by giving command for key word acute complications of hypospadias repair, wound infection in hypospadias repair, wound dehiscence in hypospadias repair, flap necrosis in hypospadias repair, edema after hypospadias repair, bleeding and hematoma in hypospadias repair, torsion after hypospadias repair, urethral stents in hypospadias repair, and fistula in hypospadias repair. Summary was reviewed of all articles and relevant articles were reviewed for full text. The information was available in acute complications in 2 articles out of 5 articles, wound infection in 10 articles out of 15 articles, wound dehiscence in 10 articles out of 15 articles, flap necrosis in 13 articles out of 5 articles, edema in 4 articles out of 9 articles, bleeding and hematoma in 5 articles out of 6 articles, penile torsion in 5 articles out of 7 articles, urethral stents in 22 articles out of 28 articles, and fistula in 27 articles out of 9430 articles. The information was common in 12 articles for more than one complication. The results were analyzed for the complications of hypospadias repairs, their causes, preventive aspect and management.

## RESULTS

The Literature in respect of acute postoperative complication is very sparse. We got about 86 articles having some mention about one or two acute complications of hypospadias repair, but as such there is not even a single article in the published English literature which deals all acute complications. Common acute complications are:

- Bleeding and hematoma
- Edema
- Wound infection
- Wound dehiscence
- Skin necrosis
- Flap necrosis
- Fistula
- Penile torsion
- Penile erections
- Inadvertent removal of urethral stent
- Bladder spasms

In order of frequency postoperative fistula is the commonest complication followed by edema and penile torsion. There were some articles on different aspects of fistula but on penile edema and torsion, few cases were mentioned. Wound dehiscence is rare but disastrous, only few cases are reported. We could find few articles on wound infection and stent problems, relevant data are discussed in individual heading.

## DISCUSSION

Complications after any surgical procedures are possible and these are higher in hypospadias surgery as compared to other reconstructive operations. The reported incidence of complications range from 6 to 30%, varying with the severity of the hypospadias.[2,3] Hypospadias repair requires delicate handling of loose and fragile tissue susceptible to edema and infection. Complications depend on the type of hypospadias, surgical technique, size of the penis, age of the child, and experience of the operating surgeon. Plate preservation procedures like tubularized incised plate (TIP) is the procedure of choice in both proximal and distal hypospadias. Fistula and flap necrosis rates are lower and the surgery more convenient with the Snodgrass urethroplasty, with better cosmetic outcome than Mathieu repair.[4] The TIP urethroplasty can be done for hypospadias reoperation provided the urethral plate is supple and previous incision of the urethral plate is not a contraindication. However, TIP repair should be avoided in repeat hypospadias surgery if the plate has been resected or is obviously scarred.[5] The TIP results are better as compared to onlay flap, both functional as well as cosmetic. Inner prepucial tube repair is technically more demanding and complications are more as compared to onlay flap repair.[6,7] The most decisive risk factor for complications is the severity of the primary malformation, because a severe malformation *per se* is difficult to treat as it requires a long reconstruction; in addition, the curvature, shortage of tissue, and extensive surgery generally require a staged reconstruction in these cases. Other factors seem to be of much lesser importance.[8] Although various techniques are similar in both pediatric age and adulthood, there is clearly a difference in terms of wound healing, infection, complication rates and overall success in adulthood. A patient undergoing surgery in adulthood should be counseled on all of these variables to avoid unreasonable expectations.[9,10] There is significant learning curve in hypospadias surgery and results improve with the experience of the surgeon.[11,12] As such results were poorer in reoperative cases[13] and free graft than in flaps,[1] but type of urinary diversion, period of urinary diversion, type of dressing, catheter size and anesthetic regime did not influence outcome significantly.[14]

## COMPLICATIONS

### Bleeding and hematoma

A significant hematoma is a potentially dangerous complication which may result in infection and/or devascularization of flaps and graft, and ultimately failure of the surgical procedure.[15] Exact incidence of hematoma is not reported in the literature, with only a few cases mentioned in individual articles. Usual cause of excessive bleeding is either bleeding from the resected corpus spongiosum for chordee correction or trauma to the corpus cavernosum or inadequate hemostasis while fashioning the skin flap. Bleeding can be minimized by dissecting in a proper plane. While dissecting the penile skin, the superficial plane is to be kept between the two layers of dartos and the deeper plane is at the level of Buck's fascia. Applying a tourniquet at the base of penis will prevent bleeding during surgery. Hematoma can be prevented by meticulous hemostasis preferably using bipolar electrocautery to minimize tissue necrosis. Fine and least reactive sutures are recommended for ligatures. Adrenaline solution (1:100,000) is also helpful for hemostasis.

Hemostasis techniques applied for maintaining a bloodless surgical field during hypospadias repair may lead to ischemia/reperfusion tissue damage in the urethral wall. Epinephrine injection may result in more prominent cellular changes compared to tourniquet techniques. However, further experimental and human studies are required to draw a firm conclusion.[16] Fibrin sealants have been used as hemostatic agent and wound healing promoters in pendulous urethroplasty.[17] Hafez and colleagues[18] reported an experimental model in rabbits using fibrin glue to patch in a defect created in the ventral tunica albuginea and evaluated at 2, 6 and 12 weeks. There were no hematomas, no evidence of corporal narrowing, and no venous leakage on cavernosography. Histopathologic evaluation demonstrated a fibrin sealant layer with angiogenesis and cellular infiltrate at 2 weeks and regeneration of normal tunica albuginea without scarring at 6 and 12 weeks.

If bleeding is still a concern, a small drain should be put in. In the postoperative period, effective external compression can temponade the bleeding but predisposes to devascularization of flap or graft. If bleeding is continued and significant, the child may need to be taken to operating room to irrigate out the accumulated blood. Such case should be evaluated for bleeding diathesis/dyscrasia.[19]

If recognized late, some of the skin sutures can be removed to allow evacuation of hematoma, along with hydrogen peroxide dressing to dissolve the adherent clots.

Excessive fibrosis (resulting from residual clots, excessive use of cautery or ligature) can result in postoperative chordee. Bleeding and hematoma is more common in adults than in pediatric age group because of frequent penile erections.

### Edema

**Figure 1:**
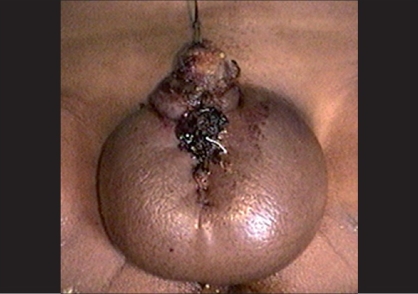
Postoperative severe edema

Postoperative edema may be excessive and may involve the penis as well as the scrotum [Figure 1]. Involvement of the meatus may result in splaying of the urinary stream but is rarely of long-term significance. Incidence of edema mentioned in the literature is about 11.11%.[20] In our experience, edema is aggravated following removal of pressure dressing usually on second to fifth postoperative day. The swelling may be aggravated by hematoma or extravasation of urine because of bladder spasm or inadvertent removal of urethral stent. Edema can be prevented by careful tissue handling, avoidance of lymphatic disconnection (by keeping the pedicle wide and minimizing tissue mobilization), use of suction drain, compressive dressing, avoiding ambulation with in 48 h in patients keeping patient recumbent position, scrotal support, and use of edema reducing medications (anti-inflammatory and serrtiopeptidase). Edema without infection or hematoma settles down with time and does not cause permanent damage. Sensory innervation of the skin influences wound healing through the release of neuropeptides from the nerve endings. Nazir *et al.*[21] reported of immunohistochemistry results, using antibodies against protein gene product (PGP) 9.5, calcitonin gene-related peptide (CGRP), and substance P (SP). The hypospadiac prepuce was found to be hypoinnervated for PGP 9.5 and CGRP positive nerves when compared with the normal prepuce (*P* < 0.05). The number of SP-positive nerves were increased in the hypospadiac prepuce, but not to statistical significance (*P* = 0.06, confidence interval >95%). There may be differences in the sensory innervation of the normal and hypospadiac prepuce. These differences in tissue environment may partly explain the postoperative edema, poor wound healing leading to urethrocutaneous fistula (UF), and increased analgesia requirement in patients undergoing hypospadias surgery.[21]

Dressing has a significant role in prevention of postoperative edema. The pressure has to adequate as excessive pressure may compromise the blood supply of flap and skin which may lead to tissue necrosis while no pressure may lead to hematoma, edema and infection increasing the incidences of complications.[22]

### Wound infection

Serious sepsis is rare, but mild and localized infection can occur because of compromised vascularity, humidity, high temperature, and proximity to a potentially contaminated area. The incidence of infection was reported very high (53%) in swabs taken from local penile meatus at admission but decreased to 30% in immediately preoperative skin swabs, following use of local preparations. Coliforms and *Staphylococcus aureus* were the most commonly grown pathogens and were sensitive to cephalosporin and aminoglycoside. Preoperative perimeatal swabs could help dictate antibiotic therapy in patients awaiting hypospadias repair.[23]

**Figure 2:**
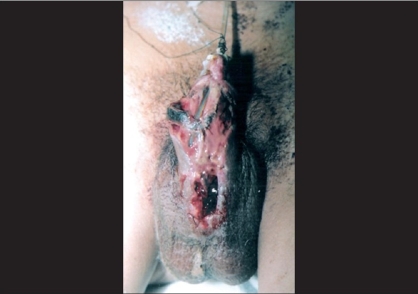
Complete dehiscence due to hematoma and infection with urethral stent *in situ* blood clot and unhealthy tissue

Infection can be a potential disaster to the repair and can be prevented by preoperative povidine iodine scrubbing, use of prophylactic antibiotics, use of antibiotic solution during surgery, avoidance of hematoma and local application of Mercurochrome.[24] Atraumatic handling of tissue, using skin hooks or stay sutures, rather than forceps is of help. If diversion is indicated, suprapubic tubes are better than intubated perineal urethrostomies. Urinary infection should be monitored during treatment. Obvious sepsis must be vigorously treated with irrigation, debridement, opening of sutures to let out suppuration, local and systemic antibiotics,[25] Severe infection may lead to disruption of repair [Figure 2] and child voiding into an open wound. In such a situation, proximal urinary diversion should be instituted. If open stent is in position and is grossly exposed, it should be removed as it will only propagate the infection. However, it can be left if there is minimal breakdown in an otherwise lengthy and healthy grafted repair to support the graft. Necrotizing fasciitis, a rapidly progressive soft tissue infection involving the skin, subcutaneous tissue and superficial fascia, is very rare in hypospadias surgery; one such case is reported.[26] Prophylactic antibiotics reduce the complications, so routine use of prophylactic antibiotics is advisable.[27,28] Postoperative urinary tract infection is rare in hypospadias repair and antibiotics are rarely needed beyond 7-10 days, but if infection persists, one should suspect a stagnation of urine in the urethra due to long/wide skin neourethra, urethral diverticula, or prostatic utricle. Urethrogram in such cases will settle the diagnosis and resection of utricle or reduction urethroplasty is done to control the infection.

### Wound dehiscence

**Figure 3:**
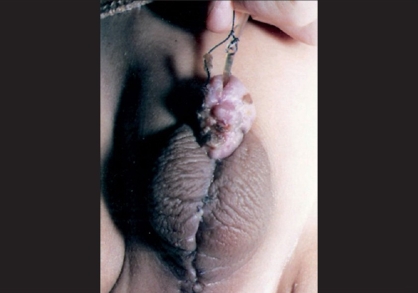
Granulation and healing after superficial skin loss without deeper tissue loss or fistula

Wound dehiscence is a very rare complication and only a few cases are reported.[29,30] Infection, edema, hematoma, erections, diminished blood supply, weakened suture material, tension at suture line, and vigorous removal of dressing may lead to wound dehiscence.[15] Wound dehiscence is more common in TIP as compared to Mathieu, but flap necrosis is more common in Mathieu.[29] Good surgical technique, apposing the dartos fascia over the urethroplasty and everting the skin edges and proper postoperative management can prevent it. Resuturing of the raw area is not advisable. Devitalized, necrotic tissue requires removal regardless of etiology of breakdown before going for any surgical repair.[19] A small raw area would granulate and re-epithelize [Figure 3], if the wound is kept clean. If the defect is large, steri-strips may be used to exert central pressure across the skin edges to encourage wound closure and minimize scarring.

### Skin and flap necrosis

**Figure 4:**
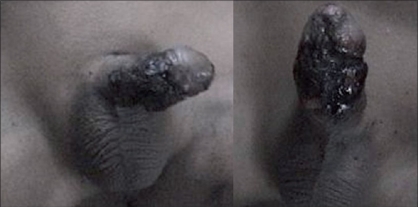
Penile skin necrosis in full thickness

**Figure 5:**
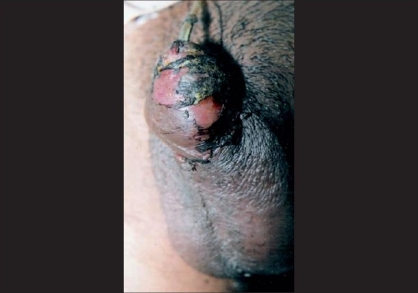
Loss of superficial layer of skin with mild edema and healthy granulation tissue below

Devascularization of the flap or graft is a major complication and reported incidence is 7%.[7,31] Flap necrosis is more common in adult hypospadias repair than in children. Various causes of devascularization are damage to vascular supply while raising the flap, hematoma, infection, vascular spasm, and tight pressure dressing [Figure 4]. The necrosis may be superficial and dermal (commonly seen due to pressure dressing) and heal without permanent damage [Figure 5]. The viability of the neourethra can be evaluated by simply looking at the outer face of the flap in case of double island flap urethroplasties.[31] It can be prevented by proper graft design, good surgical technique maintaining the proper plane of dissection, good hemostasis to avoid hematoma, administration of broad spectrum antibiotics to prevent infection, avoiding tight dressings, local application of nitroglycerin ointment to prevent vasospasm and counter incisions. If any part of graft or flap is devitalized, it should be judiciously debrided. If area of devascularization is small and pedicle of the flap is intact, satisfactory result can still be obtained without reoperating. But major dehiscence, e.g. glanular wing dehiscence in a Mathieu flip-flap repair leading to glanular breakdown, would need further operative intervention.

### Fistula

Fistula formation is most common and is often at the coronal level in tubularization urethroplasty and at the site of anastomosis in flap urethroplasty.

Beginning of fistula formation occurs early in the healing process after ventral urethral repair. Incorporation of urethral mucosa in the ventral repair is a substrate for fistula formation with rapid migration of urethral mucosa and skin epithelium into suture tracts. Whether the mucosal or dermal migration along suture tracts can be attenuated or prevented by changing the biochemical environment awaits further investigation.[32]

Incidence of fistula varies from 0[33] to 23%.[7] Incidence of fistula is less in urethral plate preservation procedures like TIP and onlay flap as compared to inner prepucial flap and tube urethroplasty.[11] The causes of fistulae remain unknown although it is likely that local infection, local ischemia and an inadequate procedure, poor tissue healing, and distal obstruction due to meatal stenosis/encrustation. Anatomical factor like severity of hypospadias and satisfaction of surgeon after surgery has significant impact on the outcome of surgery. On application of stepwise binary logistic regression, unfavorable local anatomical factors and urine leakage emerge as strong risk factors for UF and local infection as a moderate risk factor.[34] The UF rate was significantly higher in the group of patients where neourethra was constructed using 6/0 polyglactine (Vicryl) in a single layer, full-thickness, uninterrupted fashion (16.6%) compared to group of patients in which 7/0 polydioxanone (PDS) was used in the urethral anastomosis performed in a subcuticular, uninterrupted fashion (4.9%, *P* < 0.01). Use of a subcutaneous suture technique utilizing PDS suture material in urethroplasties is advocated.[35] The temptation to simply close the fistula is dangerous, as recurrent fistulae are quite common. Often it is advisable to repeat the whole urethroplasty. Urethral fistula can also be associated with a urethral stricture, which should be treated concomitantly.[36] Fibrin sealant has a unique property of hemostatic agent, urinary tract sealant and tissue adhesive. Direct injection over the anastomosis prevents urinary leakage and promotes healing. Tissue adhesives had been used with good results in urethroplasty, vesico-vaginal fistula, and hypospadias repair.[17,37] Kinahan and Johnson[38] reported using Tisseel, a fibrin glue preparation, to augment hypospadias repairs in children. When this adjunct was used, the fistula rate was lower, 9% (*n* = 78) vs. 28% (*n* = 97). Use of fibrin sealant reduces the over all complication rate in hypospadias repair.[39] Similarly use of *n*-butyl cyanoacrylate for repair of early fistula after hypospadias surgery was found effective in fistula repair. Use of this technique does not seem to affect subsequent fistula surgery,[40] but in our experience this does not help much. Various healthy and well-vascularized tissue used for prevention of fistula are dorsal/ventral dartos flap, tunica vaginalis, denuded inner prepucial skin and spongioplasty.[41,42]

A small fistula may heal spontaneously with 2-3 weeks of urinary diversion, when there is no meatal stenosis or inflammation. Spontaneous closure of fistula has been reported in up to 30% cases.[43] During this time, meatus can be dilated with an ophthalmic ointment tube tip to ensure an adequate meatal caliber. Any attempt of fistula closure at this time is of no use and should be undertaken only after 3-6 months.

### Penile torsion

**Figure 6:**
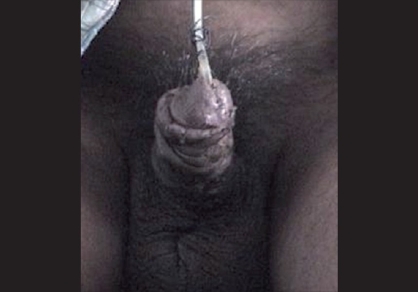
Photograph of mild penile torsion on left

Penile torsion deformity may result when onlay flap/tube repair is done, the vascular pedicle is used as second layer healthy tissue, improper closure of skin flaps, and uncorrected torsion associated with hypospadias. There are more chances of torsion in single dartos flap (mild glanular torsion 90.7% and moderate glanular torsion 9.3%) as compared to double dartos flap (0%).[42] The problem arises due to inadequate mobilization of vascular pedicle and traction on the pedicle. This can be prevented by adequate mobilization of vascular pedicle/dartos flap up to root of penis and proper adjustment of skin flaps during skin closure. Torsion of < 30° [Figure 6] does not require any corrective treatment. Moderate to severe torsion would require corrective repair at least 6 months after initial surgery. Various modalities for treatment of torsion are releasing the dorsal dartos pedicle in secondary torsion and detorque by penile degloving and realignment,[44] tunica albuginea plication,[45] suturing the tunica albuginea to pubic periosteum[46] suturing dorsal dartos opposite to torque in primary cases.[47]

### Urethral stent-related problems

**Figure 7:**
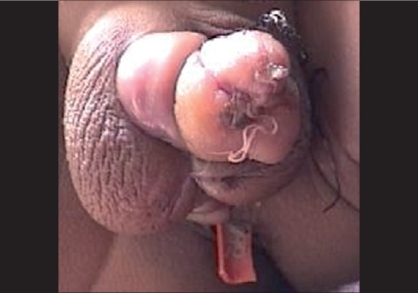
Photograph of severe edema following inadvertent removal of catheter severe hypospadias repair. Corrugated drain tube *in situ*

**Figure 8:**
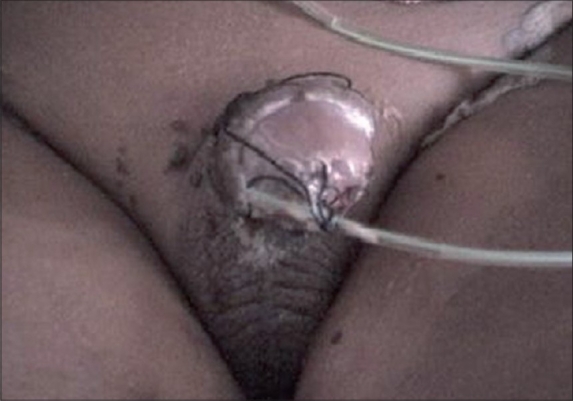
Photograph of premature pulled out urethral catheter on sixth day in distal hypospadias repair

These are catheter block, kink, inadvertent removal of catheter, and catheter knot. Catheter blockage is due to minor bleeding and inadequate hydration. Irrigation of urethral catheter with sterile normal saline can solve the problem. Rarely urethral catheter may come out prematurely leading to disastrous complications. Repositioning of urethral stent may lead to disruption of neourethra. If urethral stent has come out inadvertently within 48 h of repair [Figure 7], it is safer to put in suprapubic drainage especially in patients of flap urethroplasty or proximal hypospadias repair. But accidental removal of stent after 5 days in well-done TIP in distal hypospadias [Figure 8] may be left as such if a single gentle trial of repositioning the stent fails. Knotting is a very rare complication of urethral stent. One should suspect knotting if plane urethral stent is stuck and requires force for removal. Forceful removal of stent with knot may damage the neourethra. The mechanism of knotting appears to result from excessive intravesical catheter coiling and as the bladder decompresses the catheter tip can migrate through a coil thereby creating a knot[48] or postoperative bladder spasms may be the cause of knotting of the catheter. There are more chances of bladder spasm and knotting of catheter with longer catheterization. Over-catheterization must be avoided to prevent both knotting and troublesome bladder spasms. These points must be followed to prevent over catherization, bladder spasms, catheter knotting, and wetting of the dressing: (1) once the tube is introduced into the bladder, slowly withdraw till the urine stops dribbling (now the tip of the feeding tube lies just distal to the internal sphincter); (2) pass the tube slowly in again till urine starts to reappear (tip is just proximal to the internal sphincter); (3) push the tube in a further 2-3 cm and anchor it at this position with the glans traction suture. Besides preventing knotting this also avoids troublesome bladder spasms and straining which results in seepage of urine around the feeding tube and wetting of the dressing.[49] Knotting in supra pubic catheter had also been reported in pediatric cases that again is because of longer length of catheter in the bladder and decompression of bladder or bladder spasms.[50,51] Such impacted stents should preferably be removed by percutaneous suprapubic route. Urethral stent kinking is due to rotation of urosac, this can prevented by properly fixing the stent and urine collection bag and educating the mother to avoid tube kinking.

Other acute complications include meatal encrustation, bladder spasms, inadvertent stent removal and penile erections. Meatal encrustations can be prevented by use of urethral stents and treated with maintaining local hygiene, application of local hydrogen peroxide, and application of ophthalmic ointment tube well inside the meatus. Bladder spasms are treated with appropriate urinary diversion, repositioning of the catheter, systemic, and rarely with local intravesical anticholinergic drugs. Penile erections are common in the postoperative period. They can cause local hematoma and predisposes to infection and subsequent devascularization. These are best treated with sedation and use of ketoconazole in adults. Inadvertent removal of the stent may destroy the repair, patients of proximal hypospadias with flap or tube and dorsal inlay suprapubic diversion is indicated but distal hypospadias with TIP, urethral mobilization, or Mathieu cases may be left as such with much consequences.

## CONCLUSIONS

Fistula is the commonest complication in hypospadias repair, but healing is spontaneous in about one-third of cases provided there is no distal obstruction. Complication rate is higher in severe hypospadias and graft procedures and less in childhood surgery and plate preservation procedures. Results improve with the experience of surgeon. Little information is available on postoperative edema, bleeding and hematoma, wound dehiscence, and flap necrosis. Prospective studies are needed to know the exact incidence, causes, their impact on ultimate outcome of surgery, and suggested preventive measures for theses complications. In general unless immediate exploration is indicated for bleeding, infection or debridement, reoperation for complication should not be done before 6 months of initial surgery.[25] Overall, most of the early complications are potentially dangerous and should be better prevented. Though the literature does not project any acceptable limit of acute complications but Author's (ALB) view of acceptable limit of complications in hypospadias repair is given in Table 1. Incidence of these complications can be minimized (< 5% in distal hypospadias and < 10% in proximal hypospadias) by surgical expertise, preoperative planning, choosing appropriate surgical technique, operating in childhood, using magnification, fine suture material and judicious postoperative management.

**Table 1:** Showing acceptable limit of acute complications of hypospadias repair

Complication	Acceptable limit in distal hypospadias (%)	Acceptable limit in proximal hypospadias (%)
Bleeding and hematoma	<1	2-3
Edema	2-5	5-10
Wound infection	<1	3-5
Wound dehiscence	<1	1-2
Skin necrosis	<1	3-5
Flap necrosis	<1	3-5
Fistula	<5	<10
Penile torsion	<5	<10

## References

[1] Powel CR, Mcaleer I, Alagiri M, Kaplan GW (2000). Comparison of flaps versus grafts in proximal hypospadias surgery. J Urol.

[2] Duckett JW, Walsh PC (1998). Hypospadias. Campbell's Urology.

[3] Beuke M, Fisch M (2007). Salvage strategies after complications of hypospadias repair. Urologe A.

[4] Snodgrass WT, Lorenzo A (2002). Tubularized incised-plate urethroplasty for hypospadias reoperation. Br J Urol Int.

[5] Guo Y, Ma G, Ge Z (2004). Comparison of the Mathieu and the Snodgrass urethroplasty in distal hypospadias repair. Zhonghua Nan Ke Xue.

[6] Njinou B, Terryn F, Lorge F, Opsomer RJ, DeGroote P, Veyckemans F (1998). Correction of severe median hypospadias: Review of 77 cases treated by the onlay island flap technique. Acta Urol Belg.

[7] Elbarky (1999). A complications of the preputial island flap-tube urethroplasty. Br J Urol Int.

[8] Hansson E, Becker M, Aberg M, Svensson H (2007). Analysis of complications after repair of hypospadias. Scand J Plast Reconstr Surg Hand Surg.

[9] Hensle TW, Tennenbaum SY, Reiley EA, Pollard J (2001). Hypospadias repair in adults: Adventures and misadventures. J Urol.

[10] Dodson JL, Baird AD, Baker LA, Docimo SG, Mathews RI (2007). Outcomes of delayed hypospadias repair: Implications for decision making. Urology.

[11] Uygur MC, Unal D, Tan MO, Germiyanoglu C, Erol D (2002). Factors affecting outcome of one-stage anterior hypospadias repair: Analysis of 422 cases. Pediatr Surg Int.

[12] Castañón García-Alix M, Martín Hortigüela ME, Rodó Salas J, Morales Fochs L (1995). Complications in hypospadias repair: 20 years of experience. Cir Pediatr.

[13] Emir L, Germiyanoglu C, Erol D (2003). Onlay island flap urethroplasty: A comparative analysis of primary versus re-operative cases. Urol.

[14] Grobbelaar AO, Laing JH, Harrison DH, Sanders R (1996). Hypospadias repair: The influence of postoperative care and a patient factor on surgical morbidity. Ann Plast Surg.

[15] Elbakry A, Shamaa M, Al-Atrash G (1998). An axially vascularized meatal based flap for the repair of hypospadias. Br J Urol.

[16] Kajbafzadeh AM, Payabvash S, Tavangar SM, Salmasi AH, Sadeghi Z, Elmi A (2007). Comparison of different techniques for hemostasis in a rabbit model of hypospadias repair. J Urol.

[17] Hick EJ, Morey AF (2004). Initial experience of fibrin sealant in pendulous reconstruction: Is early catheter removal possible?. J Urol.

[18] Hafez AT, El-Assmy A, El-Hamid MA (2004). Fibrin glue for the suture-less correction of penile chordee: A pilot study in a rabbit model. BJU Int.

[19] Horton CE, Horton CE, Horton CE (1988). Complications of hypospadias surgery. Clinics in Plastic Surgery.

[20] Nonomura K, Kakizaki H, Shimoda N, Koyama T, Murakumo M, Koyanagi T (1998). Surgical repair of anterior hypospadias with fish-mouth meatus and intact prepuce based on anatomical characteristics. Eur Urol.

[21] Nazir Z, Masood R, Rehman R (2004). Sensory innervation of normal and hypospadiac prepuce: Possible implications in hypospadiology. Pediatr Surg Int.

[22] Gangopadhyay AN, Sharma S (2005). Peha-haft bandage as a new dressing for pediatric hypospadias repair. Indian J Plast Surg.

[23] Ratan SK, Sen A, Ratan J (2002). Pattern of bacterial flora in local genital skin and surgical wounds in children undergoing hypospadias repair: A preliminary study. Int J Clin Pract.

[24] Ratan SK, Sen A, Ratan J, Pandey RM (2001). Mercurochrome as an adjunct to local preoperative preparation in children undergoing hypospadias repair. Br J Urol Int.

[25] Borer JG, Retik AB, Walsh PC (2007). Hypospadias. Campbell's Urology.

[26] Luo CC, Chin Chao H, Hsun Chiu C (2005). Necrotizing fasciitis: A rare complication of hypospadias surgery in a child. J Pediatr Surg.

[27] Meir DB, Livne PM (2004). Is prophylactic antimicrobial treatment necessary after hypospadias repair?. J Urol.

[28] Lee YC, Huang CH, Chou YH, Lin CY, Wu WJ (2005). Outcome of hypospadias reoperation based on preoperative antimicrobial prophylaxis. Kaohsiung J Med Sci.

[29] Guo Y, Ma G, Ge Z (2004). Comparison of the Mathieu and the Snodgrass urethroplasty in distal hypospadias repair. Zhonghua Nan Ke Xue.

[30] Imamoğlu MA, Bakirtaş H (2003). Comparison of two methods - Mathieu and Snodgrass - in hypospadias repair. Urol Int.

[31] Chin TW, Liu CS, Wei CF (2001). Hypospadias repair using a double onlay preputial flap. Pediatr Surg Int.

[32] Edney MT, Lopes JF, Schned A, Ellsworth PI, Cendron M (2004). Time course and histology of urethrocutaneous fistula formation in a porcine model of urethral healing. Eur Urol.

[33] Kass EJ, Bolong D (1990). Single stage hypospadias reconstruction without fistula. J Urol.

[34] Ratan SK, Sen A, Pandey RM, Hans C, Roychaudhary S, Ratan J (2001). Lesser evaluated determinants of fistula formation in children with hypospadias. Int J Clin Pract.

[35] Ulman I, Erikçi V, Avanoğlu A, Gökdemir A (1997). The effect of suturing technique and material on complication rate following hypospadias repair. Eur J Pediatr Surg.

[36] Mouriquand PD, Mure PY (2004). Current concepts in hypospadiology. BJU Int.

[37] Evan LA, Ferguson KH, Foley JP, Rozanski TA, Morey AF (2003). Fibrin sealant for management of genitourinary injuries, fistulas and surgical complications. J Urol.

[38] Kinahan TJ, Johnson HW (1992). Tissel in hypospadias repair. Can J Surg.

[39] Ambriz-González G, Velázquez-Ramírez GA, García-González JL, de León-Gómez JM, Muciño-Hernández MI, González-Ojeda A (2007). Use of fibrin sealant in hypospadias surgical repair reduces the frequency of postoperative complications. Urol Int.

[40] Lapointe SP, N-Fékété C, Lortat-Jacob S (2002). Early closure of fistula after hypospadias surgery using *N*-butyl cyanoacrylate: Preliminary results. J Urol.

[41] Savanelli A, Esposito C, Settimi A (2007). A prospective randomized comparative study on the use of ventral subcutaneous flap to prevent fistulas in the Snodgrass repair for distal hypospadias. World J Urol.

[42] Kamal BA (2005). Double dartos flaps in tubularized incised plate hypospadias repair. Urology.

[43] Lay L, Zamboni WA, Texter JH, Zook EG (1995). Analysis of hypospadias and fistula repair. Am Surg.

[44] Bar-Josef Y, Binyamini J, Matzkin H, Ben-Chaim J (2007). Degloving and realignment-simple repair of isolated penile torsion. Urology.

[45] Hsieh JT, Liu SP, Chen Y, Chang HC, Yu HJ, Chen CH (2007). Correction of congenital penile curvature using modified tunical plication with absorbable sutures: The long- term outcome and patient satisfaction. Eur Urol.

[46] Zhou L, Mei H, Hwang AH, Xie HW, Hardy BE (2006). Penile torsion repair by suturing tunica albuginea to the pubic periosteum. J Pediatr Surg.

[47] Fisher C, Park M (2004). Penile torsion repair using dorsal dartos flap rotation. J Urol.

[48] Mayer E, Ankem MK, Hartanto VH, Barone JG (2002). Management of urethral catheter knot in a neonate. Can J Urol.

[49] Singh RB, Pavithran NM, Parameswaran RM (2005). Knotting of feeding tube used for bladder drainage in hypospadias repair. J Indian Assoc Pediatr Surg.

[50] Gardikis S, Solutanidis C, Deftereos S, Kambouri K, Limas C, Vaos G (2004). Suprapubic catheter knotting: An unusual complication. Int Urol Nephrol.

[51] Arda IS, Ozyaylali I (2001). An unusual complication of suprapubic catheterization with Cystofix: Catheter knotting within the bladder. Int J Urol.

